# Visualizing Noncentrosomal Microtubules during Spindle Assembly

**DOI:** 10.1371/journal.pbio.0020026

**Published:** 2004-01-20

As cells can only arise from cells that already exist, continuity of life depends on the highly regulated sequence of events that control cell division. This process is mediated by a complex macromolecular structure called the mitotic spindle. The most conspicuous components of the spindle are microtubules, which are made of tubulin and other associated proteins. In most animal cells—body cells and male germline cells (spermatocytes)—spindle assembly is orchestrated by organelles called centrosomes, which actively polymerize (that is, add tubulin subunits) and stabilize microtubules. The spindles found in these cells are known as *astral* because of the star-shaped asters—structures made of centrosome-anchored microtubules—that can be observed associating with each spindle pole. Some cells—such as the cells of the female germline (oocytes)—do not contain centrosomes, and the chromosomes themselves seem to arrange and stabilize the microtubules into spindles. These spindles are referred to as *anastral*.

To gain insight into the mechanisms of spindle assembly, scientists are increasingly relying on techniques that allow them to directly observe dynamic, complex processes in the living cell. Using time-lapse microscopy of fluorescently labeled fruitfly (Drosophila melanogaster) spermatocytes, Cayetano Gonzalez and his colleagues at the European Molecular Biology Laboratory in Germany (and now at the Centro Nacional de Investigaciones Oncológicas in Spain) have been able to observe the assembly and sorting of microtubules of noncentrosomal origin in cells that contain centrosomes. The task of flagging such microtubules is complicated by the fact that centrosomes become quite active microtubule organizers once cell division begins. Thus, as soon as the membrane around the nucleus breaks down, microtubules from the centrosome invade the nuclear region, making it hard to identify any noncentrosomal microtubules that might appear. To get around this problem, Elena Rebollo in the Gonzalez lab set up two experimental conditions under which centrosomes remain functional but are kept affixed to the cell membrane—and, therefore, away from the nucleus—in Drosophila spermatocytes. One takes advantage of a genetic mutation (called *asp*, for abnormal spindle); the other uses a transient treatment with a drug (called colcemid) that depolymerizes microtubules.

In these modified cells, microtubules can be seen growing not only over the membrane-bound centrosomes, as expected, but also over the nuclear region, away from the centrosomes. Nucleation, or formation, of such noncentrosomal microtubules has a relatively late onset, starting only once chromosomes are condensed, and takes place on the inner side of the remnants of the nuclear envelope. In a fraction of cells, these microtubules are sorted into bipolar spindle-shaped structures, highly reminiscent of the anastral spindles found in oocytes. Chromosome segregation—a critical stage of cell division—and cell division itself tend to be aberrant in these cells.

These results, Rebollo et al. propose, strongly suggest that microtubules of noncentrosomal origin may significantly contribute to spindle assembly even in cells that contain active centrosomes. Moreover, by facilitating the nucleation of such noncentrosomal microtubules, the degraded nuclear envelope may play a previously unsuspected role in spindle assembly in Drosophila spermatocytes. It is unlikely, the researchers also conclude, that the anastral spindles they have observed can fill in as a backup to ensure successful cell division. More likely, they argue, both centrosomal and noncentrosomal microtubules are required for proper spindle assembly and robust cell division in cells with centrosomes. As the authors point out, Drosophila is a rich model system that should help scientists further investigate the intricacies of spindle assembly. The answers will help us understand how the cell executes one of its most important duties: safeguarding genomic stability for future generations.

**Figure:**
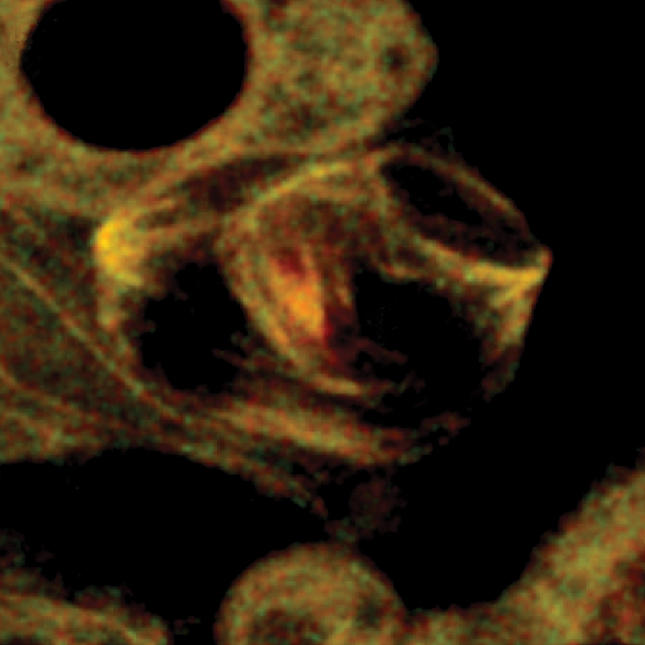
Centrosome-independent spindle assembly

